# Transoral endoscopic thyroidectomy vestibular approach (TOETVA) and complications

**DOI:** 10.1590/0100-6991e-20213084

**Published:** 2021-08-13

**Authors:** ANTONIO AUGUSTO BERTELLI, RENAN BEZERRA LIRA, ANTONIO JOSÉ GONÇALVES, LUIZ PAULO KOWALSKI

**Affiliations:** 1 - Faculdade de Ciências Médicas da Santa Casa de São Paulo, Departamento de Cirurgia - São Paulo - SP - Brasil; 2 - AC Camargo Cancer Center, Departamento de Cirurgia de Cabeça e Pescoço - São Paulo - SP - Brasil; 3 - Hospital Israelita Albert Einstein, Departamento de Cirurgia - São Paulo - SP - Brasil; 4 - Faculdade de Medicina da Universidade de São Paulo, Departamento de Cirurgia - São Paulo - SP - Brasil

O The review article from Menderico Jr and colleagues^1^, a systematic review on Transoral Endoscopic Thyroidectomy Vestibular Approach (TOETVA) and its complications, discusses this thyroidectomy technique that has been studied and used in several Asian, European, and American countries. TOETVA uses common laparoscopy instruments and three portals through the inferior buccal vestibule, a space between the inferior dental arch and the lip. The technique has been shown to be reproducible in several centers, and has a short learning curve, between 10 and 15 cases^2^. Proofs of this are the 11 papers presented during the XXVII Brazilian Congress of Head and Neck Surgery, in 2019, by six different groups in Brazil^3^, one of which was accepted to compete for the Jorge Fairbanks Barbosa^2^ ^,^ ^3^ award, the most important in the specialty.

We read the cited article very carefully, and despite the search strategy, said to be broad, between the years 2015 and 2020, the authors selected no article from the year 2020^1^. As this is a recent technique, many articles will certainly bring higher complication rates, as they study the beginning of each group’s casuistry and often the learning curve of each surgeon. It is important to highlight that the authors report having selected six articles after the criteria adopted by the systematic review, but only five studies comprise the two tables of results^1^. Of these five items, four have 10 cases or less^1^, evidencing initial sample sizes. A systematic review on the same topic, published in 2018, included 16 articles (of which 14 were on TOETVA) and 785 patients^4^. It is noteworthy that the article by Menderico Jr et al., two years later, comprised only five articles and 459 patients^1^, as well as the fact that the authors did not consult any expert in the technique, a common practice in systematic reviews of surgical nature^4^.

In 2019, Bertelli et al. published an article about the beginning of their experience in a teaching hospital with the first 15 cases, showing 33% of postoperative complications, one mental nerve paresthesia, one inferior laryngeal nerve palsy, one hypoparathyroidism and two skin burns^2^. Although this study evaluated the presence of complications during the first author’s learning curve, no permanent complications were observed: the mental nerve paresthesia occurred only in the first patient in the series, and resolved spontaneously within four months of the postoperative period; the vocal fold paralysis lasted for two months; the transient hypoparathyroidism required calcium supplementation for 40 days; and both skin burns were minor, without flap perforation, and evolved without aesthetic consequences^2^.

In early 2020, De Cicco et al. published a study comparing 31 patients undergoing TOETVA with 30 patients submitted to conventional surgery^5^. The authors also detailed postoperative complications, comparing them between groups, and demonstrated that TOETVA is safe for selected patients^5^.

Also in 2020, Lira et al. published their initial series of 56 TOETVA procedures, assessing complications, operative time, and learning curve, comparing them with a control group of 745 open operations^6^. In the largest Brazilian series published to date, they showed no significant difference in complications after TOETVA when compared with open surgery^6^, especially regarding the risk of infection, contrary to what Menderico Jr et al. claim in the conclusion of their review article^1^. In the 2018 article by Anuwong et al., included in the review, with 425 patients undergoing TOETVA, there was not a single case of surgical site infection^7^. In Table 2 of the article by Menderico Jr et al., which listed the complications found in the five selected articles^1^, we were unable to identify the information that led to this wrong conclusion.

The works from Bertelli et al.^2^, De Cicco et al.^5^, and Lira et al.^6^ represent Brazilian samples, and unfortunately none of these were included in the systematic review by Menderico Jr et al.^1^.

At least two more systematic reviews were published on the subject, one in 2018 and another in 2019^8^ ^,^ ^9^, in addition to the one mentioned above, also from 2018^4^. All included more than 10 articles, with more than 700 patients, and concluded that TOETVA is a safe technique for selected patients, without evidence that the risk of contamination or infection of the surgical site is higher than with open thyroidectomy. It is also important to point out that the most recent systematic reviews exclude series with less than ten cases^6^, which would leave the study in question only with the article by Anuwong et al.^7^. Although the Menderico Jr et al. article’s Methods section states that “systematic review articles were used for discussion of the results”^1^, the three articles referred to herein^4^ ^,^ ^8^ ^,^ ^9^ were not mentioned, nor any other systematic reviews between the references.

In a brief search carried out only in the PubMed database, we identified 14 articles published prior to the acceptance date of the review by Menderico Jr et al. All have data on complications, with more than 10 cases, totaling 858 patients, as recorded in Table 1. Eleven of these were not even mentioned, three were mentioned, but the data were not included, making it clear that the systematic review by Menderico Jr et al. did not review all relevant literature.

**Table 1:** Articles on TOETVA that include data on complications published before the date of acceptance of the systematic review by Menderico Jr et al. (Source: Pubmed).

Author	Journal	Year	Volume	n°	
Park et al.	Surg Endoscop	2019	33(9):3034-3039	15	Not quoted
Luna-Ortiz et al.	Ann Surg Oncol	2020	27(5):1356-1360	46	Not quoted
Jitpratoom et al.	Gland Surg	2016	5(6):546-552	46	Not included in the review
Russell et al.	Laryngoscope Investig Otolaryngol	2018	24;3(5):409-414	20	Not included in the review
Pérez-Soto et al.	J Laparoendosc Adv Surg Tech A	2019	29(12):1526-1531	20	Not included in the review
Sun et al	Surg Endosc	2020	34(1):268-274	100	Not quoted
Fernandez Ranvier et al.	J Laparoendosc Adv Surg Tech A	2020	30(3):278-283	152	Not quoted
Kim et al.	Surg Endosc	2020	34(12):5414-5420	132	Not quoted
Yi et al.	Ann Surg Treat Res	2018	95(2):73-79	20	Not quoted
Luo et al.	J Laparoendosc Adv Surg Tech A	2020	30(2):163-169	204	Not quoted
Chen et al.^8^	Eur Arch Otorhinolaryngol	2019	276(2):297-304	SR* (864)	Not quoted
Razavi et al.	Head Neck	2018	40(10):2246-2253	27	Not quoted
Park et al	Surg Endosc	2019	33(7):2104-2113	65	Not quoted
Sivakumar et al.	J Minim Access Surg	2018	14(2):118-123	11	Not quoted
Total**				858	
	*systematic review				
	** excluding systematic review				

Therefore, it seems to us that the systematic review by Menderico Jr et al. presents an unacceptable sequence of methodological biases, from the selection of articles to interpretations with biases in personal opinions, which culminates in the mistaken conclusion that there is a greater risk of infection related to TOETVA. As transoral thyroidectomy is a new technique and many data on the subject are being published, including national ones, we recommend caution when interpreting the study published by Menderico Jr et al., due to an evident failure in the inclusion and interpretation of published works.
